# Uncovering the dynamics of enterprises digital transformation research: A comparative review on literature before and after the COVID-19 pandemic

**DOI:** 10.1016/j.heliyon.2024.e26986

**Published:** 2024-02-28

**Authors:** Jinnan Wu, Xinyi Qu, Linghui Sheng, Wentao Chu

**Affiliations:** School of Business, Anhui University of Technology, Ma’anshan, Anhui, 243032, China

**Keywords:** Digital transformation, COVID-19 pandemic, Uncertainty, Dynamic capabilities, CiteSpace

## Abstract

The COVID-19 pandemic has greatly changed global practices of enterprise digital transformation (EDT). However, the impact of the pandemic on EDT research patterns remains unexplored. This study examines the overall development and research pattern shift of literature on EDT in the field of business and economics. A bibliometric analysis with CiteSpace was conducted on a total of 140 journal articles indexed the SSCI and SCIE databases on Web of Science prior to the pandemic and 621 articles published after the pandemic. The results suggest that following the outbreak of COVID-19 pandemic, there has been a significantly rapid growth of EDT-related publications, and the contributing role in EDT research of influential countries has undergone significant changes. Furthermore, the changes in keyword patterns were identified before and after the pandemic. Specifically, EDT research after the COVID-19 outbreak has been focusing on emerging topics, such as corporate governance, sustainable development, platform ecosystems, and dynamic capabilities. Finally, recommendations for future research are provided at individual, organizational, and ecosystem levels. Overall, this study is one of the first studies to uncover the dynamics of EDT research patterns due to the COVID-19 Pandemic, thus enhancing our understanding of the features and structures of digital transformation research in uncertain environment.

## 1 Introduction

The Corona Virus Disease 2019 (COVID-19) pandemic has increased the “black swan” incidents, posing a greater challenge to global enterprises to survive and even grow in the face of adversity and uncertainty. Facing the increasingly complex and volatile global environment caused by the pandemic, enterprise digital transformation (EDT) has become a strategic choice that global enterprises must make [1]. According to recent research by International Data Corporation (IDC), EDT investments are generating more than 5% revenue growth for 85% of Chinese enterprises and more than 10% revenue growth for 48% of Chinese enterprises, with total spending on digital transformation in China reaching $2.38 trillion from 2022 to 2026.^1^ The COVID-19 pandemic has not only forced enterprises to undergo digital transformation to change strategic and operational models, also changed the direction of EDT research for businesses. Due to lockdown measures and home quarantine, the COVID-19 pandemic has posed unprecedented challenges to global businesses in terms of increased market uncertainty, supply chain disruptions, remote work mandates, and consumer habit changes [[1], [2], [3]]. These challenges motivate researchers pay more attention to understand how digital transformation can be implemented to support employees’ remote work arrangements [2], increase reliance on e-commerce [4], create digital customer engagement [3], enhance analytics capabilities for real-time insights [5], and foster adaptable business models and resilient supply chains [3,6]. Thus, a comparative review on literature before and after the COVID-19 pandemic enable us to uncover the dynamics of EDT research and identify future directions, and can offer critical insights for current digital transformation initiatives of businesses.

Despite gaining significant momentum in recent years, the academic attention towards EDT dates back two decades. Since Andal-Ancion et al. [7] paid early attention to the role of new information technologies in accomplishing strategic changes among traditional businesses twenty years ago, researchers from various disciplines have increasingly recognized the strategic importance of EDT. EDT represents a significant shift for traditional business, which includes the integration and utilization of relevant digital technologies, aiming to innovate and reshape their existing business models [8,9]. It encompasses a comprehensive restructuring of organizations supported by information systems, accompanied by fundamental economic and technological changes at both the organizational and industrial levels [10,11]. EDT involves the changes that digital technologies may bring to enterprises' products, organizational structures or process automation, which in turn change their overall business models [8,9,12]. Over recent years (especially since the COVID-19 pandemic in 2020), scholars have conducted numerous studies on the concepts and connotations, influencing factors, and their business value of EDT, with fruitful research results. Hence, a scientific and systematic analysis of the research progress and hot issues in this field, especially for revealing the research hotspots and trends after the pandemic, will not only help to uncover new research directions, but also provide practical insights for improving successful EDT. Quantitative advantages of bibliometric method can overcome the limitations of subjective human judgement [13]. The appendix reports eight review articles on EDT using bibliometric methods. Although these studies have provided valuable insights into our understanding of digital transformation research published before 2022, some theoretical gaps should be further addressed. First, there is an absence of research contrasting the topic shifts in digital transformation research before and after the COVID-19 pandemic. Second, the majority of literature review has concentrated on specific industries [14,15], issues [16,17], or discipline [18,19], with only two exceptions [13,20]. Third, while most studies conducted keyword co-occurrence analysis, only one study undertook keyword clustering to identify the focal themes within digital transformation research [15]. Lastly, almost all reviews have not covered the latest publications on EDT in the past year, which is not conducive to revealing the latest research trends in this field.

This article differs from above reviews as it strictly focuses on the knowledge gap regarding the changes in number of publications, influential countries, highly cited publications, and research hotspots before and after the COVID-19 pandemic. More importantly, this article looks for characteristics of EDT research after the pandemic, which may provide valuable insights into how enterprises adapted to unprecedented changes. To bridge the gaps and gain insights into the overall development and shifting focus of EDT research caused by the pandemic, this study utilizes bibliometric methods and visualization tools to address the following research questions (RQ).

RQ1 What changes have occurred in annual tendency of EDT research, influential countries and regions, and high cited papers before and after the pandemic?

RQ2 What changes have occurred in the core keywords and keywords features of EDT research before and after the pandemic?

RQ3 What can we learn from the topic shifts of EDT research due to the impact of pandemic?

In doing so, this study makes contributions to digital transformation research in four ways. First, a detailed assessment of annual publication and citation trends in EDT literature will be conducted, providing an in-depth evaluation of its growth over time. Furthermore, we offer valuable insights into the most productive sources, countries or regions, and highly cited publications, both before and after the onset of the pandemic. Second, the study will employ co-citation networks to explore influential sources and references within EDT research. This approach will highlight academic relationships and connections before and after the pandemic, shedding light on the evolution of EDT research. Third, a keyword perspective will be employed to uncover research themes by analyzing co-occurrence degrees in different keyword clusters. This approach allows us to identify research topics in both pre- and post-pandemic literature, while also providing insights into emerging trends in the field following the COVID-19 pandemic. Lastly, considering the dynamic nature of EDT research in the pandemic context, the study will discuss potential avenues for future research at the individual, organizational, and societal levels. By addressing these research objectives, this study will contribute to the overall understanding of EDT research, provide valuable insights into its development, and offer guidance for future research endeavors.

The paper is organized as follows. Section 2 presents the data source and analysis strategies. In Sections 3, we will present the findings of primary analysis, performance analysis, co-citations analysis, co-occurrence analysis, and conceptual structure analysis. Section 4 will discuss future research directions. The study will be concluded in Section 5.

## 2 Methods

### 2.1 Data source

To explore changes in the characteristics of the EDT literature before and after the COVID-19 pandemic, we referred to the existing review literature [13,20,21] to design a data collection and screening process. The original dataset was collected from Web of Sciences Core Collection in the Science Citation Index Expanded (SCIE) and Social Sciences Citation Index (SSCI) databases. Consistent with the purpose of this study, the subject terms were limited to “digital transformation”, “digital technology”, “digital technologies”, “digital strategy”, “digital strategies”, “digital business transformation”, and “digitalization”. The time span was set from 2003, when Andal-Ancion et al. [7] introduced digital transformation to the field of business administration, to October 2023. A total of 15,062 journal articles within the SSCI and SCIE were obtained. Then, the initial dataset needed to be screened to determine the data valid for further analysis, considering discipline, title, abstract, and keywords. This screening process aimed to ensure that only appropriate data were selected for subsequent analysis.

**Fig. 1:**
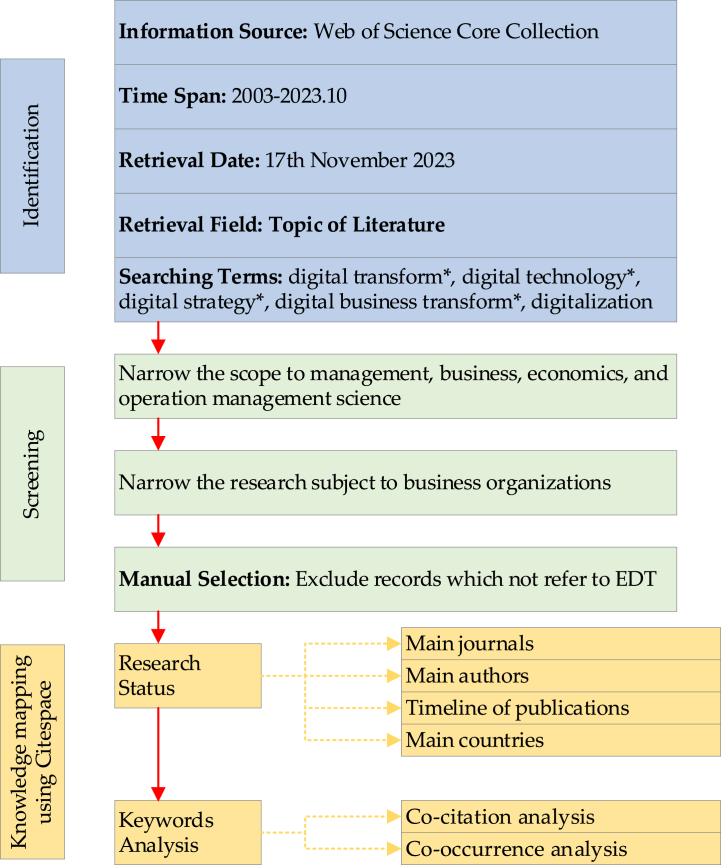
Diagram of the employed methodology.

As for the specific screening process, the original dataset was initially screened to ensure that the publications were selected from business and economics such as management, business, economics, and operation management science since our main focus is on the mechanisms that affect EDT from a management perspective, and 2791 papers were retained. We then proceeded to verify the relevance of the publications to business organizations by conducting a manual review of their titles and abstracts. Any publications that did not primarily focus on business organizations were excluded from the dataset. Following this filtering process, we eliminated papers with low correlation, resulting in a dataset consisting of 761 publications. Finally, in line with Boulanger [22], we set January 2020 as the line of demarcation between two final datasets. The first dataset includes 140 pre-pandemic publications and the second datasets includes 621 post-pandemic publications. The summary of data source and selection is displayed in Fig. 1.

### 2.2 Bibliometric analyses

Bibliometrics offers a significant advantage by allowing to explore specific research fields through the analysis of citations, co-citations, geographical distribution, and keyword frequency to capture its evolutionary process [23]. The CiteSpace tool uses literature data to visualize the evolutionary history within a knowledge domain, identify research frontiers through the citation and co-citation clusters represented on the knowledge map [24]. In this study, the CiteSpace tool was utilized to answer the first two critical research questions. More specifically, we used it to transform sample publications information into scientific knowledge with special format, which was then analyzed to reveal the changes occurred in annual tendency of EDT research, influential countries and regions, and high cited papers before and after the pandemic (RQ1). Following this, the co-citation analysis, co-occurrence analysis, and cluster analysis were performed to identify the changes occurred in the core keywords and keywords features of EDT research before and after the pandemic (RQ2). Lastly, grounded in the findings of RQ2, we provided future recommendations from the perspectives of the individual, organization, and ecosystem (RQ2).

## 3 Results

### 3.1 Primary analysis

**Fig. 2:**
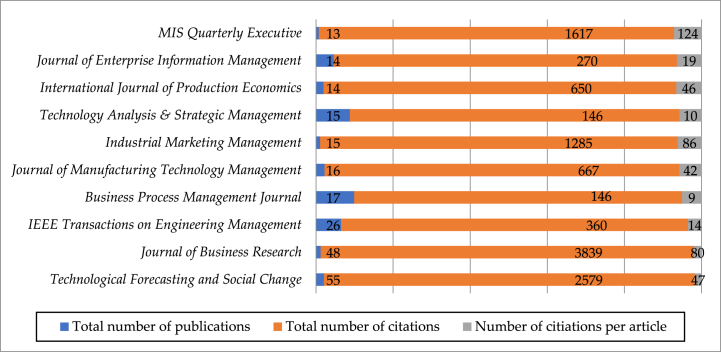
Total amount and citations and average citations of EDT publications in Top 10 journals.

In order to identify the productive sources related to EDT research, the volume and citation frequency of journals publishing 761 papers were analyzed with CiteSpace. Fig. 2 presents top ten academic journals publishing EDT research: *Technological Forecasting and Social Change(55), Journal of Business Research(48), IEEE Transactions on Engineering Management(26), Business Process Management Journal(17), Journal of Manufacturing Technology Management(16), Technology Analysis & Strategic Management(15), Industrial Marketing Management(15), International Journal of Production Economics(14), Journal of Enterprise Information Management(14), and MIS Quarterly Executive(13)*. Overall, top ten academic journals (less than 5%) have published a total of 233 papers (more than 30%), indicating that they are the most important journals publishing EDT research. Among all journals, *Technological Forecasting and Social Change* and *Journal of Business Research* are the most dominant journals which have published a total of 103 papers (13.5% of the sample publications). Significantly, although *MIS Quarterly*, a top journal in the field of information management, published only 9 papers, they were cited 3009 times with an average citation of 334 times, far larger than the second ranked *MIS Quarterly Executive* with an average citation of 124 times, suggesting that *MIS Quarterly* is the most cited journals on EDT research.

**Fig. 3:**
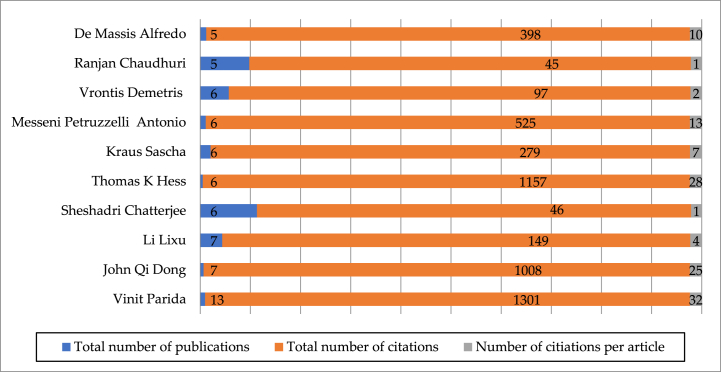
Total amount and citations and average citations of EDT publications in Top 10 authors.

To further identify influential researchers in the field of EDT research, we computed the total publications and citations of 2042 researchers across 761 publications. The findings are presented in Fig. 3, highlighting the most published researchers are Vinit Parida (13), John Qi Dong (7), Li Lixu (7), Sheshadri Chatterjee (6), Thomas K Hess (6), Kraus Sascha (6), Messeni Petruzzelli Antonio (6), Vrontis Demetris (6), Ranjan Chaudhuri (5) and De Massis Alfredo (5). However, not all high-published researchers are high-cited ones. Among these ten EDT researchers, the most cited ones are Vinit Parida with 1301 citations, Thomas K Hess with 1157 citations, John Qi Dong with 1008 citations, Messeni Petruzzelli Antonio with 525 citations, De Massis Alfredo with 398 citations, Kraus Sascha with 279 citations, Li, Lixu with 149 citations, Vrontis Demetris with 97 citations, Sheshadri Chatterjee with 46 citations, and Ranjan Chaudhuri with 45 citations. Overall, Vinit Parida, John Qi Dong, and Thomas K Hess are the most influential researchers in the field of EDT research.

### 3.2 Performance analysis

**Fig. 4:**
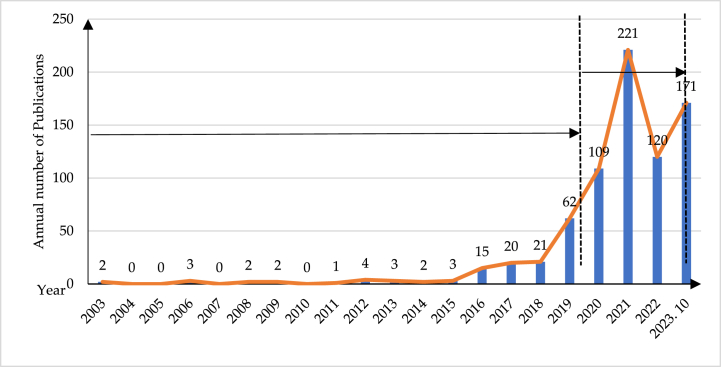
Publications distribution of EDT research over the years.

Fig. 4 illustrates the trajectory of global research publications on EDT from 2003 to October 2023, revealing a consistent upward trend in article numbers overtime. Notably, there was a substantial increase from 140 articles published prior to the outbreak of COVID-19 pandemic to 621 articles published afterwards. Before the pandemic, the research on EDT is still in its initial and exploratory stage, with an average annual number of 8 articles, indicating that it has not yet attracted enough attention from scholars of related disciplines. The COVID-19 pandemic that occurred in 2020 has caused a huge shock to enterprises around the world, and it is more urgent than ever for enterprises to explore ways to respond. Digital transformation, meanwhile, is considered as one of the most important strategies to enhance organizational resilience to cope with the uncertain environment. In response to the needs of management practices, scholars in the fields of various disciplines (e.g., information management, strategic management and economics) have begun to focus on EDT. Thus, there has been a noticeable surge in research activity in EDT area，and has a peak in 2021 (221 publications). The average annual count of post-pandemic publications is 162, representing a significant exponential growth from the pre-pandemic average of 8 per year, indicating that the research on EDT after the pandemic is highly valued by global scholars.

**Fig. 5:**
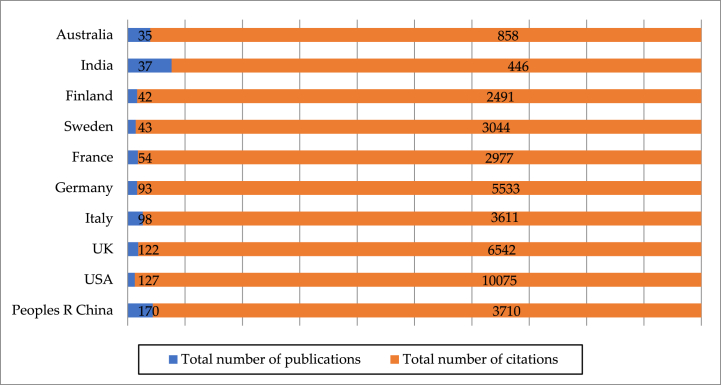
Total amount and citations of EDT publications in Top 10 Countries.

The country and region visualization identifies 72 countries and regions worldwide from 761 publications, with the majority located in Europe, Asia, and the Americas. Fig. 5 provides the top ten influential countries which play an important role in EDT research from 2003 to October 2023. Among them, the U.S. has been the leader in EDT research with 127 publications (ranking the second) and 10,075 total citations (ranking the first). The PR China makes important contribution to EDT research with the most publications (170) among all countries, but ranks the fourth with a total of 3710 citations. Moreover, three European countries, namely the UK, Germany, and Italy, hold a leading position in terms of both the total publications and citations.

To further explore the changes in the geographical distribution of scholars, Table 1 presents the total number of publications and citations for the top ten influential countries and regions in EDT research during 2003–2019 and 2020–October 2023. Prior to the COVID-19 pandemic, the U.S. is fully ahead of other countries in two indicators (31 and 5744). Germany (19 and 3205) and the UK (18 and 2584) closely follow the United States in total number of publications and citations. However, it should be pointed out that PR China ranks the seventh in total number of publications (5), but it ranks fifth in total number of citations (1302), suggesting that it has played an increasingly important role in this field.

**Table 1:** Top 10 countries based on total publications and citations.

Country	pre-pandemic		post-pandemic		Growth rate	
	Total number of publications	Total number of citations	Total number of publications	Total number of citations	Total number of publications	Total number of citations
P R China	5	1302	165	2408	3200%	85%
USA	31	5744	96	4331	210%	−25%
UK	18	2584	104	3958	478%	53%
Italy	9	503	89	3108	889%	518%
Germany	19	3205	74	2328	289%	−27%
France	5	519	49	2458	880%	374%
Sweden	12	1918	31	1126	158%	−41%
Finland	11	1245	31	1246	182%	0%
India	0	0	37	443	–	–
Australia	4	106	31	752	675%	609%

After the COVID-19 pandemic, the total number of publications has increased dramatically in all countries. It is worth noting that we have witnessed a rapid increase in the academic impact of Chinese scholars in this field after the pandemic. The total number of their publications has risen from 5 before the pandemic to 165 after the pandemic (a growth rate of 3200%), which is the highest among all countries. The United States maintains its leading advantage in terms of total citations (4331). However, its total number of publications (96) has dropped to the third place, and its growth rates in total number of publications and citations (210% and −25%) are lower than most other countries, respectively. Moreover, we found that the total number of publications from Italy, France, India, and Australia experienced a rapid growth after the pandemic. Meanwhile, Italy, France, and China hold a significant advantage in both total number of citations and growth rate of total citations, indicating their increasingly significant academic impact in this field.

### 3.3 Co-citation analysis

A higher citation frequency indicates greater academic value and influence for a paper [25]. To clarify the dynamic characteristics of EDT research before and after the pandemic, we conducted separate co-citation analyses for pre- pandemic and post-pandemic EDT publications. The findings presented in Table 2 show the top ten high cited literatures before the pandemic, primarily focusing on the opportunities that digital technologies bring to enterprises and the challenges they face in EDT process.

**Table 2:** Top 10 cited EDT-related publications before the COVID_19 pandemic.

Article titles and authors	Sources	Total citations	Annual citations
Understanding Digital Transformation: A Review and A Research Agenda (Vial, 2019)	*The Journal of Strategic Information Systems*	1305	270
Digital Business Strategy: Toward a Next Generation of Insights (Bharadwaj et al., 2013)	*MIS Quarterly*	1214	155
Digital Innovation Management: Reinventing Innovation Management Research in a Digital World (Nambisan et al., 2017)	*MIS Quarterly*	988	258
Digital Entrepreneurship: Toward a Digital Technology Perspective of Entrepreneurship (Nambisan, 2017)	*Entrepreneurship Theory and Practice*	883	230
Building Dynamic Capabilities for Digital Transformation: An Ongoing Process of Strategic Renewal (Warner et al., 2019)	*Long Range Planning*	647	134
The Digital Transformation of Innovation and Entrepreneurship: Progress, Challenges and Key Themes (Nambisan et al., 2019)	*Research Policy*	643	133
Options for Formulating A Digital Transformation Strategy (Hess et al., 2016)	*MIS Quarterly Executive*	577	119
Driving Forces and Barriers of Industry 4.0: Do Multinational and Small and Medium-Sized Companies Have Equal Opportunities? (Horváth et al., 2019)	*Technological Forecasting and Social Change*	441	91
Digital Innovation and Transformation: An Institutional Perspective (Hinings et al., 2018)	*Information and Organization*	417	147
Digital Marketing: A Framework, Review and Research Agenda (Kannan and Li, 2017)	*International Journal of Research in Marketing*	408	106

Before the COVID-19 pandemic, the publication that exerted the most influence in the field of EDT was “Understanding Digital Transformation: A Review and A Research Agenda*”* contributed by Vial [26], published in *The Journal of Strategic Information Systems*. This paper received a total of 1305 citations, with an annual citation count of 270, significantly surpassing the second-most cited publication. In this study, Vial [26] reviewed and summarized the definitions and theories of digital transformation, and proposed dynamic capabilities and ethical issues as an important direction for future research on digital transformation strategies. The second-most cited document was published by Bharadwaj et al. [27] in *MIS Quarterly*, with the total citation of 1214 and annual citation of 155. Drawing on both resource-based view and dynamic capability view, Bharadwaj et al. [27] examined business IT strategy and identified four key themes of digital business strategy: scope, scale, speed, and sources of business value. Their work facilitated a comprehensive discussion on the role of digital resources in the emerging industry ecosystem, which significantly advances our understanding of digitalization and provides valuable insights for future research. The other most cited pre-pandemic EDT publications are Nambisan et al. [28] with 988 citations, Nambisan [29] with 883 citations, Warner and Wäger [30] with 647 citations, Nambisan et al. [31]with 643 citations, Hess et al. [8] with 577 citations, Horváth and Szabó [32] with 441 citations, Hinings et al. [33] with 417 citations, and Kannan and Li [34] with 408 citations. Overall, pre-pandemic EDT literature focuses on the theoretical concept of digital transformation and its business value created for individual enterprises, but pays less attention to antecedents of digital transformation and the underlying mechanisms.

Table 3 presents the top ten publications related to EDT that received the highest citations after the COVID-19 pandemic. The most influential EDT paper, titled “*Digital Transformation: A Multidisciplinary Reflection and Research Agenda*”*,* was published by Verhoef et al. [35] in *Journal of Business Research*, garnering the largest total and annual citations (821 and 290). The authors proposed three stages of digital transformation: *digitization, digitalization,* and *digital transformation*. They also identify and delineate growth strategies for digital firms as well as the assets and capabilities required in order to successfully transform digitally. The other most cited pre-pandemic EDT publications are Hanelt et al. [36] with 365 citations, Belhadi et al. [37] with 320 citations, Matarazzo et al. [38] with 251 citations, Kohtamäki et al. [39] with 229 citations, Ritter and Pedersen [40] with 213 citations, Correani et al. [41] and Amankwah-Amoah et al. [42] with 173 citations, Mora Cortez and Johnston [43] with 172 citations, and Tortorella et al. [44] with 168 citations. Overall, post-pandemic EDT research focuses on how digital technologies disrupt traditional survival models and how internal and external environmental changes enable EDT during COVID_19 pandemics.

**Table 3:** Top 10 cited EDT-related publications after the COVID_19 pandemic.

Article titles and authors	Sources	Total citations	Annual citations
Digital Transformation: A Multidisciplinary Reflection and Research Agenda (Verhoef et al., 2021)	*Journal of Business Research*	821	290
A Systematic Review of The Literature on Digital Transformation: Insights and Implications for Strategy and Organizational Change (Hanelt et al., 2021)	*Journal of Management Studies*	365	129
Manufacturing and Service Supply Chain Resilience to the Covid-19 Outbreak: Lessons Learned From the Automobile and Airline Industries (Belhadi et al., 2021)	*Technological Forecasting and Social Change*	320	113
Digital Transformation and Customer Value Creation in Made in Italy Smes: A Dynamic Capabilities Perspective (Matarazzo et al., 2021)	*Journal of Business Research*	251	89
The Relationship Between Digitalization and Servitization: The Role of Servitization in Capturing the Financial Potential of Digitalization (Kohtamäki et al., 2020)	*Technological Forecasting and Social Change*	229	60
Digitization Capability and the Digitalization of Business Models In Business-to-Business Firms: Past, Present, and Future (Ritter and Pedersen, 2020)	Industrial Marketing Management	213	56
Implementing a Digital Strategy: Learning from the Experience of Three Digital Transformation Projects (Correani et al., 2020)	California Management Review	173	45
Covid-19 and Digitalization: The Great Acceleration (Amankwah-Amoah et al., 2021)	Journal of Business Research	173	61
The Coronavirus Crisis in B2B Settings: Crisis Uniqueness and Managerial Implications Based on Social Exchange Theory (Cortez and Johnston, 2020)	Industrial Marketing Management	172	45
Organizational Learning Paths Based upon Industry 4.0 Adoption: An Empirical Study with Brazilian Manufacturers (Tortorella et al., 2020)	International Journal of Production Economics	168	44

Unlike pre-pandemic literature, post- pandemic EDT research pays more attention to the drivers and resistance caused by environmental factors to successful EDT [35,41], and discusses more about the business values brought by digital transformation to supply chain and business ecosystems outside focal organization [37,38]. For example, more EDT research has focused on Industry 4.0 [44], supply chain resilience [37], and strategy change [36,41].

### 3.4 Co-occurrence analysis

**Fig. 6:**
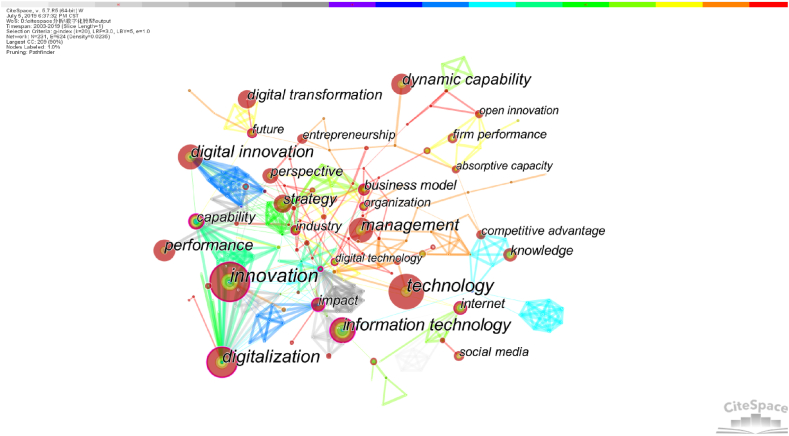
Keywords co-occurrence network of pre-pandemic EDT literature.

The research hotspots in a particular field can be revealed by examining the keywords found in the literature over an extended period [45]. Using CiteSpace, high-frequency keywords were extracted respectively before and after the pandemic, and keyword co-occurrence analysis was performed to further capture the focus shift within EDT research. Firstly, a keyword co-occurrence analysis was conducted for pre-pandemic publications. The node type was set as keywords, and the selection criteria queue was based on the g-index with a threshold of 20. The time period considered was before the pandemic (2003.01–2019.12), with each slice representing one year. To obtain the pre-pandemic EDT research network, the network was pruned with pathfinder. Fig. 6 illustrates this network, where each node represents a keyword. The size of the node represents the frequency of the keyword, while the thickness and color of the connecting line indicates the closeness of the connections and their changes over time, respectively.

The results indicate that the keywords that appear frequently in pre-pandemic publications are “innovation”, “technology”, “information technology”, “digital innovation”, “management”, " digitalization”, “performance”, “dynamic capability” and “strategy”. The keywords with high network centrality include “innovation”, “competition”, “digitalization”, “impact”, “information technology”, “internet”, “capability” and “digital business strategy”. The dense connection between “innovation”, “capability” and “digitalization” indicates that pre-pandemic publications focus on how to build capabilities through digital transformation for enterprises to achieve improved innovation performance. The more intense linkage between “impact” and “information technology” suggests that scholars pay more attention to whether and how information technology affects business operations and innovation activities.

**Fig. 7:**
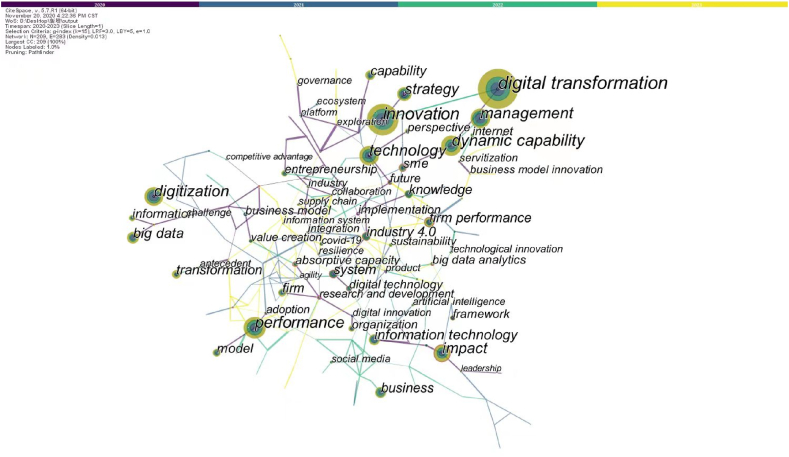
Keywords co-occurrence network of post-pandemic EDT literature.

With the same parameters, the keyword co-occurrence network of post-pandemic EDT publications was presented in Fig. 7. Beside the same high-frequency keywords appearing in pre-pandemic literature, some newly emerging keywords that appear frequently in post-pandemic literature are “digital transformation”, “impact”, “big data”, “system”, “industry 4.0″, and “knowledge”. Similarly, new keywords with high network centrality include “implementation”, “research and development ", “big data analytics”, “absorptive capacity”, “adoption”, “crisis” and “collaboration”. Given that the impact of the COVID-19 pandemic on SMEs is the greatest in all countries, researchers are increasingly concerning about why SMEs accept big data and related information systems to enable digital transformation. Also, more academic attentions have been paid to create and seize new opportunities of improving interorganizational collaboration and knowledge sharing.

Comparing the keyword co-occurrence plots before and after the COVID-19 pandemic, we find that there are more keyword connections and higher network density before the pandemic, which indicates that EDT research focuses on more similar issues. However, more keyword nodes were witnessed in post-pandemic literature, suggesting that the COVID-19 pandemic has to some extent stimulated the emergence of new research topics and the continuous innovation of valuable research themes.

Before the COVID-19 pandemic, significant academic endeavors were dedicated to understanding how businesses could attain value through EDT, thereby reshaping their operations and enhancing their products and services [26,27]. Researchers focused on EDT strategies and their effects on established business strategies, thus aiding enterprises in realizing business value of digital transformation. However, after the pandemic, there has been a shift in researchers’ attention from individual enterprises to encompassing multiple participants, such as the supply chain and business ecosystems [46]. This shift is driven by the need for increased collaboration with external partners among businesses, particularly SMEs and new start-ups, because more effort is needed to recover from losses within an environment of growing uncertainty. Moreover, digital technology has the potential to alleviate the adverse effects of pandemic prevention measures by overcoming temporal and geographic barriers, which subsequently facilitates stronger connections among stakeholders such as enterprises, consumers, and business partners [2].

### 3.5 Conceptual structure analysis

To identify the current research hotspots of EDT more scientifically and clearly, a cluster analysis of keywords was further conducted to demonstrate structural features between keyword clusters and highlight the key nodes. Initially, the keywords from 140 pre- pandemic literatures were clustered with CiteSpace. This process yielded 10 clusters, as shown in Table 4, with a modularity (Q) of 0.7549, indicating a significant cluster structure. Additionally, a silhouette value of 0.8979 confirmed the confidence of the clustering results.

**Table 4:** Top 10 keyword clusters in terms of size before the COVID_19 pandemic.

Ranking	Size	Silhouette	Cluster labeling	Time scope	High frequency keywords
#0	27	0.946	digitalization	2006–2019	network-of-pattern; management; complexity et al.
#1	25	0.818	firm performance	2009–2019	creative; sourcing; ambidexterity; product idea et al.
#2	23	0.813	disruptive technologies	2009–2019	evolution; database; creative collaboration et al.
#3	19	0.907	C2C	2009–2014	price service; competition; industry policy et al.
#4	17	0.718	innovation process	2017–2019	growth; product definition; sustainability et al.
#5	17	0.920	strategic alignment	2003–2019	user experience; evolution; collaboration et al.
#6	16	0.992	firm	2009–2012	automotive industry; knowledge management; digital innovation et al.
#7	16	0.978	digital business strategy	2013–2018	competitive advantage; management; business strategy et al.
#8	15	0.848	service innovation	2013–2019	technology; information system; value creation et al.
#9	12	1.000	start-ups	2003–2017	internet; social media; co-creation et al.

**Fig. 8:**
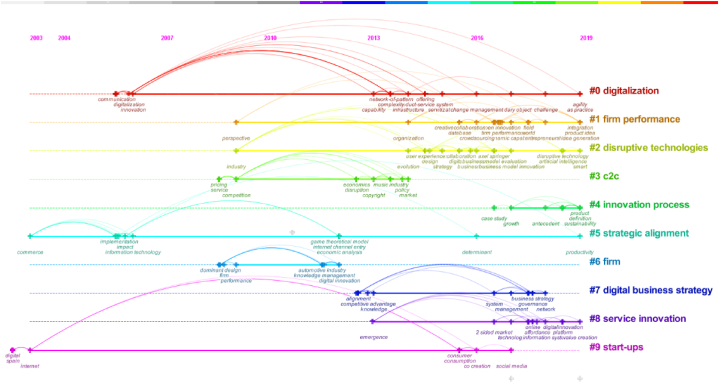
Mapping keyword clustering timeline of pre-pandemic EDT literature.

Based on the cluster mapping, the timeline diagram of keyword clustering was created to showcase the relationships and time spans between clusters. This visual representation allows for the observation of the trend of each cluster over time, facilitating a greater understanding of the dynamic evolution of the research hotspots. Fig. 8 illustrates the keyword clustering timeline diagram before the COVID-19 pandemic. The first subsequent section provides detailed descriptions of the first six keyword clusters.

#### 3.5.1 Cluster#0: digitalization

This category represents the central focus of EDT research, spanning from 2006 to 2019. It examines the use of digital technologies and organizational management innovation in the context of EDT. The critical objective is to explore how enterprises can efficiently adopt and utilize emerging digital technologies to transform themselves and thus enhance their competitiveness. For example, Liang Li et al. [47] argued that information technology change plays a crucial role in driving EDT. Singh and Hess [48] suggested that enterprises can leverage digital technologies to achieve significant improvements in business operations and organizational changes, thereby participating in a broader ecosystem to maintain their competitive edge.

#### 3.5.2 Cluster#1: firm performance

Research in this category, which spans from 2009 to 2019, focuses on the effects of digital transformation on business innovation, product conception, supplier sourcing, and ambidextrous strategies. For example, Lerch and Gotsch [49] found that as the trend towards digitalization continues, manufactures are improving their services by utilizing digital systems to improves performance and efficiency. Dubey et al. [50] confirmed that digital technologies enable efficient integration and allocation of existing resources, facilitates the upgrading of products and services, and accelerates firm innovation.

#### 3.5.3 Cluster#2: disruptive technologies

This research category centers on the value-creating effects of foundational digital technologies such as artificial intelligence, blockchain, cloud computing, and big data, during the period from 2009 to 2019. The development of disruptive technologies produces a profound transformation in the organizational structure of enterprises, industry frameworks, and even the underlying economic paradigm. Moreover, disruptive technologies play a pivotal role in platform management and contribute to the ecological development of enterprise [51]. Alcácer and Cruz-Machado [52] emphasized that Industry 4.0 leads the digital age where factories achieve real-time engagement of all elements of the value chain with the help of digital technology at a higher level of automation, which has a disruptive impact on manufacturing companies.

#### 3.5.4 Cluster#3: C2C

This category of research, covering the period from 2009 to 2014, focuses on price competition in C2C e-commerce and the associated industrial policies, as well as the impact of C2C on consumers, enterprises, and markets. For instance, Feng et al. [53] conducted a study on the optimal distribution channel structure for digital content, such as video, images and music, in order to explore the business value of both B2C and C2C channels simultaneously.

#### 3.5.5 Cluster#4: innovation process

Focusing on the years 2017–2019, this research category explores the role of digital transformation in driving enterprises to reinvent their products and attain value growth and sustainability. Nambisan et al. [28] raised critical questions about the fundamental assumptions regarding the relationship between innovation processes and outcomes. They shed light on the disruptive potential of the rapid and extensive digitalization of innovation processes and outcomes, which can challenge existing theories of innovation management. Additionally, Oh et al. [54] found that information technology can enhance enterprises’ ability to integrate resources, thereby fostering positive relationship with customers and improving overall performance.

#### 3.5.6 Cluster#5: strategic alignment

This category spans from 2003 to 2019, highlighting its prominence in EDT research. Strategy alignment emphasizes that the realization of the value-creating potential of digital transformation significantly depends on the degree of alignment between digital technologies and the core business of the enterprise. Bharadwaj et al. [27] argued that digital technologies have the capacity to fundamentally reshape business strategies, making the achievement of alignment between digital and business strategies which is a fundamental driver for creating and capturing business value. Drnevich and Croson [55] further pointed out that digital technologies can influence the range of strategic options and value creation opportunities available to enterprises, helping them with better capabilities to plan, forecast, and model consumer behavior, complying with government regulations, and make other decisions.

The same method was employed to cluster the keywords from 621 post- pandemic literatures. Initially, 7 clusters were captured, and then the mapping was adjusted based on objective data to ensure accuracy. Then, 7 clusters were formed, as presented in Table 5. The modularity (Q) was 0.7461, and the silhouette was 0.8903, indicating a significant clustering structure and providing credible clustering results.

**Table 5:** Top 10 keyword clusters in terms of size after the COVID_19 pandemic.

Ranking	Size	Silhouette	Cluster labeling	Time scope	High frequency keywords
#0	24	0.813	corporate governance	2020–2023.10	resource-based view; internationalization; corporate digitalization et al.
#1	23	0.91	sustainable development	2020–2023.10	resilience; sustainability; digital strategy consensus et al.
#2	21	0.942	platform ecosystems	2020–2023.10	supply chain resilience; big data analytics; industry 4.0 technology et al.
#3	16	0.952	digital-related capabilities	2020–2023.10	risk management; digital awareness of managers; efficiency et al.
#4	16	0.851	technological innovation	2020–2023.10	creation; production improvement; digital skill et al.
#5	14	0.923	dynamic capabilities	2020–2023.10	organizational ambidexterity; agility; external knowledge et al.
#6	13	0.897	entrepreneurial orientation	2020–2023.10	market orientation; digitization; digital infrastructure et al.

**Fig. 9:**
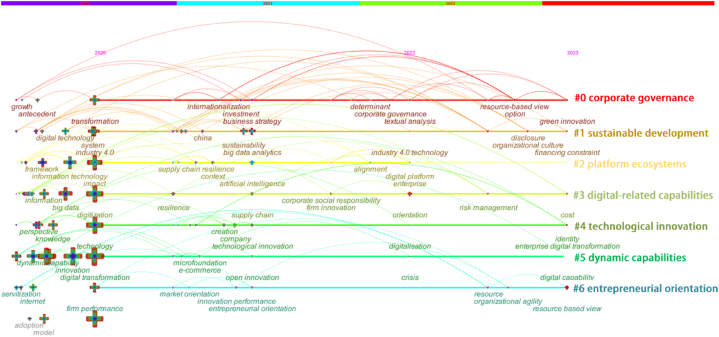
Mapping keyword clustering timeline of post-pandemic EDT literature.

The findings in Table 5 indicate that “digitalization” and “innovation” remain prominent themes in EDT research even after the pandemic. However, the global business environment was significantly affected by the sudden crisis of the COVID-19 pandemic, which resulted in the emergence of new and increasingly popular research topics in EDT research. Furthermore, a timeline mapping analysis was conducted for post-pandemic keywords using the same methodology. The timeline diagram in Fig. 9 depicts the results of keywords clustering after the COVID-19 pandemic. Compared to the pre-pandemic period, four new keywords have emerged in the post-pandemic period as the focus of current research on enterprise digital transformation: “corporate governance”, “sustainable development”, “platform ecosystems” and “dynamic capabilities”. These keywords highlight the shifting landscape of research priorities in the field. In order to provide a more comprehensive understanding, the following paragraphs will describe the five keyword clusters with larger clustering sizes in greater detail.

#### 3.5.7 Cluster#0: corporate governance

Governance is a system of incentives and controls. Organizations with effective corporate governance not only have a significant impact on their performance, but are also critical to their growth and long-term survival [56]. In the context of the COVID-19 pandemic, the business environment has radically changed, and the mechanisms for interaction between enterprises and stakeholders have been put to the test [57], causing significant changes to corporate governance. The principle of sustainable development is also in the spotlight during the pandemic, when enterprises are required to ensure business continuity as a primary objective to gain robustness for survival and thriving. In addition, digitalization is changing the work habits of employees and the way organizations operate. Enterprises depend on the innovative technologies and additional information that digitalization brings to maintain their competitiveness and differentiate themselves from other companies, which has led researchers to pay closer attention to the positive role that digitalization can play in the field of corporate governance.

#### 3.5.8 Cluster#1: sustainable development

The COVID-19 pandemic has exacerbated the uncertainty in the current business environment and exposed the vulnerability of traditional enterprises in the face of adversity, which demonstrates the importance of sustainable development. Researchers have been paying close attention to the opportunities and threats posed by the pandemic, which is a typical emergent crisis event to business development, as well as its role in driving EDT processes. Scholars have primarily focused on exploring how enterprises can leverage digital transformation to increase organizational resilience and gain competitive advantage in a crisis similar to the COVID-19 pandemic, and ultimately achieving the goal of sustainable development. For instance, Soto-Acosta [4] proposed that traditional enterprises affected by the novel coronavirus outbreak should consider utilizing digital technologies to address operational and business disruptions caused by social isolation, ultimately bolstering organizational resilience in the face of turbulent external environment. Rozak et al. [58] found that enterprises need a more valuable strategy to adapt to great changes, making sustainability in the post pandemic era become more challenging.

#### 3.5.9 Cluster#2: platform ecosystems

With the emergence of platform-based ecosystems, the interconnections between different stakeholders are becoming stronger [59]. Research in this area focuses not only on how a single enterprise can diversify through friendly exchanges with complementary external networks, but also on how to govern the construction of platforms for the effective management of the various players in the ecosystem. With the involvement of digital technologies, these participants co-create value and synergize under the coordination of platforms, distinguishing themselves from traditional organizations by their source of power, motivation, nature of incentives, governance and coordination structures [60]. Simultaneously, digital platforms, by their geographically oblivious nature, have flourished in the context of the enforced social isolation brought about by the pandemic. Based on the digital technologies provided by the platform owners, the ecosystem participants contribute to the development of the overall productive capacity through their individual innovative capabilities [31], with benefits and governance issues that follow.

#### 3.5.10 Cluster#5: dynamic capabilities

Dynamic capability refers to the ability of enterprises to rapidly respond to environmental changes [61]. In an increasingly complex and uncertain environment, enterprises can sustain their core competitive capabilities in the emerging digital economy by developing robust dynamic capabilities that enable swift creation, implementation, and transformation of business models [62]. In essence, the COVID-19 pandemic served as a catalyst, highlighting the critical importance of embracing digital transformation. Given the significant uncertainty caused by the COVID-19 pandemic in business operations, researchers have been exploring ways to reshape business strategies through digital transformation, with the purpose to enhance the agility of enterprises and their supply chains, facilitating their recovery, sustainability, and potential improvement in overall business performance [3]. Characteristics such as ambidexterity and agility have also received much attention from scholars as drivers that may have a positive impact on shaping dynamic capabilities. Enterprises with dynamic capabilities were better equipped to navigate uncertainties, adapt to changes, foster innovation, and achieve resilience and sustainable growth in a rapidly evolving landscape.

When comparing the keyword clusters before and after the pandemic, it becomes apparent that certain clusters, such as “digitalization” and “digital-related capabilities”, “start-ups” and “entrepreneurial orientation”, as well as “disruptive technologies”, “innovation process” and “technological innovation”, remain the same or show similarities. These topics have consistently been of great interest to scholars researching EDT, and their relevance is not directly tied to the emergence of the COVID-19 pandemic. However, despite spanning the COVID-19 pandemic period, the specific research questions have significantly evolved due to the growing understanding of EDT and the continuously changing business environment. For instance, the pre-pandemic “digitalization” cluster covers core keywords such as “network model”, “management”, and “complexity”, whereas the post-pandemic cluster of “digital-related capabilities” shifted its emphasis to “digital awareness of managers”, “efficiency”, and “business-to-business (B2B)". Similarly, the pre-pandemic cluster of “start-ups” centered on keywords such as “Internet”, “social media”, and “co-creation”, whereas the post-pandemic cluster of “entrepreneurial orientation” incorporated new core keywords like “servitization”, “strategic orientation”, and “digital infrastructure”.

## 4 Future research directions

Based on our co-occurrence analysis and clustering analysis, current EDT research can be categorized into three levels: individual, organizational, and ecosystem. Extant literature has undertaken varying degree of explorations into the antecedents and outcomes of EDT at these three levels. These research efforts have not only provided insights into the topics that have been widely addressed but have also highlighted areas that merit further investigation. In this section, we will discuss future directions in EDT research based on the above-mentioned results of co-occurrence and clustering analysis at the individual, organizational, and ecosystem levels.

### 4.1 Individual level

Prior research on EDT primarily focuses on its impacts on organizations, supply chains, and business ecosystems at organizational and inter-organizational levels. However, an organizational ability to achieve their intended strategic goals through digital transformation largely depends on the adaptation of employees to the challenges brought by EDT [5]. However, both pre- and post-pandemic literature has predominantly focused on the outcomes of EDT at organizational and industrial levels. In contrast, limited attention has been paid to employees’ psychological and behavioral responses to EDT. Given the fact that successful EDT largely depends on how employees accept and adapt to changes in work content and job skills brought by EDT [63], future research will benefit from exploring potential positive or negative impacts of EDT on employees. For instance, while mandatory telecommuting during the COVID-19 pandemic allows workers to overcome spatial constraints, it can also disrupt work routines and productivity [64]. Hence, future research should explore the positive effects of EDT on employees' work performance and psychological well-being, as well as investigate the underlying mechanism involved. Furthermore, it is crucial to identify potential negative effects and design effective intervention mechanisms to mitigate them.

### 4.2 Organizational level

Despite existing EDT research offering insights into our understanding of whether and how it affects business innovation (*cluster #4 of pre-pandemic publications*) and performance (*cluster #1 of pre-pandemic publications*), more research is needed to deepen our understanding of other organizational impacts of digital transformation [65,66], for example, internal control performance, relationship performance, environmental performance, ethical performance, and consumer behavioral responses [67,68]. Despite considerable progress in understanding the positive effects, there is room for revealing why many EDT projects fail to achieve anticipated strategic objectives. Actually, numerous enterprises have undergone a decline in performance after implementing digital transformation strategies, with some even facing the risk of collapse [64]. Hence, More efforts are need to examine potential negative effects imposed by EDT on business [69], thus enhancing our understanding of the risks and costs associated with digital transformation. Another valuable research direction is to identify the critical failure factors of EDT initiatives, as the current body of research predominantly focuses on its outcomes. To capture expected organizational outcomes of digital transformation strategies, researchers have identified critical success factors of EDT such as improving strategic alignment between technology and business (*cluster #5 of pre-pandemic publications*), strengthening corporate governance through incentives and controls (*cluster #0 of post-pandemic publications*), and fostering dynamic capabilities for strategic and organizational changes (*cluster #5 of post-pandemic publications*). Undoubtedly, these endeavors are important; however, analyzing those failure cases of digital transformation to identify critical factors contributing to such failures can help other enterprises avoid risks and costs. This is equally, if not more, crucial than comprehending critical success factors.

### 4.3 Ecosystem level

Due to the joint effects of technological innovation, uncertain environment, and the COVID-19 pandemic, platforms and ecosystems have emerged as pivotal organizational forms for attaining sustainable development in the digital era [70]. EDT plays a significant role in facilitating the formation or participation of platform ecosystems, fostering stronger ties and trust among business partners [71,72]. Thus, more recent EDT research pays attention to platform ecosystems (*cluster #2 of post-pandemic publications*). Prior study has established that digital transformation enables enterprises to leverage external resources to achieve borderless development [73,74], and to benefit from knowledge and experience spillovers generated by their partners [73,75,76]. However, the literature exploring how digital transformation contributes to ecosystem remains limited [77]. In this context, further research endeavors are recommended to address the following questions: when an enterprise should participate platform ecosystems? How enterprises manage and coordinate various stakeholders on platforms, including suppliers, partners, and users? What governance models contribute to platform ecosystems’ stability and innovation? How to ensure that partners within certain ecosystem utilize business data belonging to other stakeholders in a compliant rather than malicious manner? By answering these questions, we can reveal how enterprises effectively use digital technologies to construct or engage with platform ecosystems. This, in turn, increases organizational resilience and achieves sustainable development through optimizing internal and external resources allocation and fostering inter-organizational knowledge learning.

## 5 Conclusion and limitations

By employing CiteSpace, a bibliometric and visual analysis tool, we thoroughly conducted a scientific and quantitative analysis on substantial publications to compare the research landscape and highly cited works on EDT before and after the COVID-19 pandemic. Through various analyses including performance, co-citation, co-occurrence, and cluster analysis, we identified significant changes resulting from the pandemic. Firstly, the COVID-19 pandemic had a profound impact on enterprises worldwide, which leads to global research interest in EDT after 2020 by creating unpredictable challenges such as market uncertainty, supply chain disruptions, remote work, and changing consumer habits caused by the pandemic worldwide [[1], [2], [3]]. Secondly, Although the United States remained the leading contributor to publications both pre- and post-pandemic, Chinese scholars experienced a significant increase in the international visibility of their EDT research following the COVID-19 pandemic. Chinese researchers play increasingly impactful contributions to global EDT research due to this country’s successful pandemic response, technological prowess, industrial shifts, and active international collaboration. Thirdly, prior to the pandemic, research on EDT had not yet formed a comprehensive theoretical framework, focusing on defining digital concepts and constructing research frameworks. However, after the pandemic, EDT research has made substantial progress, expanding its scope to include corporate governance, sustainable development, platform ecosystems and dynamic capabilities domains. Such topics shift of EDT research is global researchers' active responses to help businesses worldwide enhance organizational and supply chain resilience and achieve sustainable development under the sudden impact of the COVID-19 pandemic [3,6,58,62]. Lastly, our findings reveal that three central themes consistently paly pivotal roles in current EDT research, i.e., “digital technology” (as a “tool”), “innovation” (as a “goal”) and “entrepreneurship” (as a “trend”). These themes continue to shape the trajectory of research in this field, irrespective of the effects of the pandemic. However, in response to the challenges posed by the COVID-19 outbreak, enterprises have faced the urgent need to navigate the complexities of the external environment, which has led to a focus on fostering collaborations with external partners and acquiring critical resource support. Consequently, several new research themes, such as “corporate governance,” “sustainable development,” “platform ecosystems,” and “dynamic capabilities”, have emerged as significant areas of interest.

Overall, this study, through a bibliometric approach, provides a comprehensive and better understanding of EDT-related research observed before and after the COVID-19 pandemic. Multiple shifts and changes in this field are identified, allowing for a better understanding of the features and structures of EDT research in the realm of business and economics. This analysis highlights the evolving nature of research in response to the pandemic and offers insights into the trends and themes that have emerged in the post-COVID-19 landscape. Our review has some limitations. First, potential bias in the selected articles may challenge our findings. Although it is a general rule in bibliometrics, searching only in peer-reviewed academic journals may miss some relevant studies included in books or papers. However, through a rigorous and well-documented search process, we collected and analyzed as complete a review sample as possible. Second, in the intricate tapestry of digital transformation, the use of visualization tools highlights specific connections while potentially ignoring others. Specifically, critics may point to a lack of analytical depth in each keyword cluster. However, despite the confined scope of our observations, we contend that our extensive descriptions of each topic add value to progress in this area. This ought to serve as a foundational framework for forthcoming research endeavors aimed at delving deeper into characterizing the diverse sub-studies of digital transformation within an uncertain environment.

## Funding statement

This work was supported by the 10.13039/501100001809National Natural Science Foundation of China (Grant No. 72172002) and Philosophical and Social Science Key Foundation of Anhui Province (Grant No. AHSKZ2020D19).

## Data availability statement

The datasets used in this research are available upon request from the corresponding author.

## Additional information

No additional information is available for this paper.

## CRediT authorship contribution statement

**Jinnan Wu:** Writing – review & editing, Validation, Supervision, Project administration, Investigation, Funding acquisition, Formal analysis, Conceptualization. **Xinyi Qu:** Writing – original draft, Visualization, Software, Methodology, Formal analysis, Data curation, Conceptualization. **Linghui Sheng:** Software, Methodology, Data curation. **Wentao Chu:** Software, Data curation.

## Declaration of competing interest

The authors declare that they have no known competing financial interests or personal relationships that could have appeared to influence the work reported in this paper.

## Figures and Tables

**Table:** Review articles employing bibliometric methodologies

Publications	Timespan	Specific sector or issue	Clustering analysis	Comparison before and after COVID_19
Pizzi et al. (2021)	1985–2020	Impacts of digital transformation on internal auditing	No	No
Zhu et al. (2021)	2000–2020	No	Cited reference clustering	No
Kraus et al. (2022)	2010–2020	Digital transformation in business and management research	No	No
Guandalini (2022)	2000–2021.8	Sustainability through digital transformation	No	No
Shi et al. (2022)	1999–2021.8	No	No	No
Lalic et al. (2022)	2001–2022.8	Digital Transformation in the Engineering Research	Keywords clustering	No
Savkovic et al. (2022)	2006–2022.8	Digital transformation in manufacturing sector	No	No
Calderon-Monge and Ribeiro-Soriano (2023)	2018–2022	The role of digitalization in business and management	No	No

## Footnotes

1 International Data Corporation (IDC): China Digital Transformation Market Forecast, 2021–2026: Practicing Digital Priority Strategy through Application Scenarios.

## References

[1] Reuschl A.J., Deist M.K., Maalaoui A. (2022). Digital transformation during a pandemic: stretching the organizational elasticity. J. Bus. Res..

[2] Autio E., Mudambi R., Yoo Y. (2021). Digitalization and globalization in a turbulent world: centrifugal and centripetal forces. Global Strat. J..

[3] Khurana I., Dutta D.K., Singh Ghura A. (2022). SMEs and digital transformation during a crisis: the emergence of resilience as a second-order dynamic capability in an entrepreneurial ecosystem. J. Bus. Res..

[4] Soto-Acosta P. (2020). COVID-19 pandemic: shifting digital transformation to a high-speed gear. Inf. Syst. Manag..

[5] Trenerry B., Chng S., Wang Y., Suhaila Z.S., Lim S.S., Lu H.Y., Oh P.H. (2021). Preparing workplaces for digital transformation: an integrative review and framework of multi-level factors. Front. Psychol..

[6] Priyono A., Moin A., Putri V.N. (2020). Identifying digital transformation paths in the business model of SMEs during the COVID-19 pandemic. J. Open Innov. Technol. Market Compl..

[7] Andal-Ancion A., Cartwright P.A., Yip G.S. (2003). The digital transformation of traditional business. MIT Sloan Manag. Rev..

[8] Hess T., Matt C., Benlian A., Wiesböck F. (2016). Options for formulating a digital transformation strategy. MIS Q. Exec..

[9] Majchrzak A., Markus M.L., Wareham J. (2016). Designing for digital transformation lessons for information systems research from the study of ICT and societal challenges. MIS Q..

[10] Besson P., Rowel F. (2012). Strategizing information systems-enabled organizational transformation: a transdisciplinary review and new directions. J. Strat. Inf. Syst..

[11] Karimi J., Walter Z. (2015). The role of dynamic capabilities in responding to digital disruption: a factor-based study of the newspaper industry. J. Manag. Inf. Syst..

[12] Hai T.N., Van Q.N., Thi Tuyet M.N. (2021). Digital transformation: opportunities and challenges for leaders in the emerging countries in response to covid-19 pandemic. Emerg. Sci. J..

[13] Zhu X., Ge S., Wang N. (2021). Digital transformation: a systematic literature review. Comput. Ind. Eng..

[14] Savkovic M., Lalic D.C., Lalic B., Miloradov M., Curcic J., Simeunovic N., Kim D.Y., von Cieminski G., Romero D. (2022). Advances in Production Management Systems. Smart Manufacturing and Logistics Systems: Turning Ideas into Action.

[15] Lalic D.C., Gracanin D., Lolic T., Lalic B., Simeunovic N., Kim D.Y., von Cieminski G., Romero D. (2022). Advances in Production Management Systems, Smart Manufacturing and Logistics Systems: Turning Ideas into Action.

[16] Pizzi S., Venturelli A., Variale M., Macario G.P. (2021). Assessing the impacts of digital transformation on internal auditing: a bibliometric analysis. Technol. Soc..

[17] Guandalini I. (2022). Sustainability through digital transformation: a systematic literature review for research guidance. J. Bus. Res..

[18] Calderon-Monge E., Ribeiro-Soriano D. (2023). The role of digitalization in business and management: a systematic literature review. Rev. Managerial Sci..

[19] Kraus S., Durst S., Ferreira J.J., Veiga P., Kailer N., Weinmann A. (2022). Digital transformation in business and management research: an overview of the current status quo. Int. J. Inf. Manag..

[20] Shi L., Mai Y.P., Wu Y.J. (2022). Digital transformation: a bibliometric analysis. J. Organ. End User Comput..

[21] Xu X.R., Hou G.M., Wang J.P. (2022). Research on digital transformation based on complex systems: visualization of knowledge maps and construction of a theoretical framework. Sustainability.

[22] Boulanger S.O.M. (2022). The roadmap to smart cities: a bibliometric literature review on smart cities' trends before and after the COVID-19 pandemic. Energies.

[23] Liao H.C., Tang M., Luo L., Li C.Y., Chiclana F., Zeng X.J. (2018). A bibliometric analysis and visualization of medical big data research. Sustainability.

[24] Cobo M.J., López-Herrera A.G., Herrera-Viedma E., Herrera F. (2011). Science mapping software tools: review, analysis, and cooperative study among tools. J. Am. Soc. Inf. Sci. Technol..

[25] Moed H.F. (2002). The impact-factors debate: the ISI's uses and limits. Nature.

[26] Vial G. (2019). Understanding digital transformation: a review and a research agenda. J. Strat. Inf. Syst..

[27] Bharadwaj A., El Sawy O.A., Pavlou P.A., Venkatraman N. (2013). Digital business strategy: toward a next generation of insights. MIS Q..

[28] Nambisan S., Lyytinen K., Majchrzak A., Song M. (2017). Digital innovation management: reinventing innovation management research in a digital world. MIS Q..

[29] Nambisan S. (2017). Digital entrepreneurship: toward a digital technology perspective of entrepreneurship. Entrep. Theory Pract..

[30] Warner K.S.R., Wäger M. (2019). Building dynamic capabilities for digital transformation: an ongoing process of strategic renewal. Long. Range Plan..

[31] Nambisan S., Wright M., Feldman M. (2019). The digital transformation of innovation and entrepreneurship: progress, challenges and key themes. Res. Pol..

[32] Horváth D., Szabó R.Z. (2019). Driving forces and barriers of Industry 4.0: do multinational and small and medium-sized companies have equal opportunities?. Technol. Forecast. Soc. Change.

[33] Hinings B., Gegenhuber T., Greenwood R. (2018). Digital innovation and transformation: an institutional perspective. Inform. Org..

[34] Kannan P.K., Li H.A. (2017). Digital marketing: a framework, review and research agenda. Int. J. Res. Market..

[35] Verhoef P.C., Broekhuizen T., Bart Y., Bhattacharya A., Qi Dong J., Fabian N., Haenlein M. (2021). Digital transformation: a multidisciplinary reflection and research agenda. J. Bus. Res..

[36] Hanelt A., Bohnsack R., Marz D., Antunes C. (2021). A systematic review of the literature on digital transformation: insights and implications for strategy and organizational change. J. Manag. Stud..

[37] Belhadi A., Kamble S., Jabbour C.J.C., Gunasekaran A., Ndubisi N.O., Venkatesh M. (2021). Manufacturing and service supply chain resilience to the COVID-19 outbreak: lessons learned from the automobile and airline industries. Technol. Forecast. Soc. Change.

[38] Matarazzo M., Penco L., Profumo G., Quaglia R. (2021). Digital transformation and customer value creation in Made in Italy SMEs: a dynamic capabilities perspective. J. Bus. Res..

[39] Kohtamäki M., Parida V., Patel P.C., Gebauer H. (2020). The relationship between digitalization and servitization: the role of servitization in capturing the financial potential of digitalization. Technol. Forecast. Soc. Change.

[40] Ritter T., Pedersen C.L. (2020). Digitization capability and the digitalization of business models in business-to-business firms: past, present, and future. Ind. Market. Manag..

[41] Correani A., Massis A.D., Frattini F., Petruzzelli A.M., Natalicchio A. (2020). Implementing a digital strategy: learning from the experience of three digital transformation projects. Calif. Manag. Rev..

[42] Amankwah-Amoah J., Khan Z., Wood G., Knight G. (2021). COVID-19 and digitalization: the great acceleration. J. Bus. Res..

[43] Mora Cortez R., Johnston W.J. (2020). The Coronavirus crisis in B2B settings: crisis uniqueness and managerial implications based on social exchange theory. Ind. Market. Manag..

[44] Tortorella G.L., Cawley Vergara A.M., Garza-Reyes J.A., Sawhney R. (2020). Organizational learning paths based upon industry 4.0 adoption: an empirical study with Brazilian manufacturers. Int. J. Prod. Econ..

[45] Onan A., Korukoglu S., Bulut H. (2016). Ensemble of keyword extraction methods and classifiers in text classification. Expert Syst. Appl..

[46] Li F.M., Long J.C., Zhao W. (2023). Mining braces of innovation linking to digital transformation grounded in TOE framework. Sustainability.

[47] Liang Li, Su Fang, Zhang Wei (2018). Digital transformation by SME entrepreneurs: a capability perspective. Inf. Syst. J..

[48] Singh A., Hess T. (2017). How chief digital officers promote the digital transformation of their companies. MIS Q. Exec..

[49] Lerch C., Gotsch M. (2015). Digitalized product-service systems in manufacturing firms A case study analysis. Res. Technol. Manag..

[50] Dubey R., Gunasekaran A., Childe S.J., Papadopoulos T., Luo Z., Wamba S.F., Roubaud D. (2019). Can big data and predictive analytics improve social and environmental sustainability?. Technol. Forecast. Soc. Change.

[51] Yoo Y., Boland R., Lyytinen K., Majchrzak A. (2012). Organizing for innovation in the digitized world. Organ. Sci..

[52] Alcácer V., Cruz-Machado V. (2019). Scanning the industry 4.0: a literature review on technologies for manufacturing systems. Eng. Sci. Technol. Int. J..

[53] Feng Y.F., Guo Z.L., Chiang W.Y.K. (2009). Optimal digital content distribution strategy in the presence of the consumer-to-consumer channel. J. Manag. Inf. Syst..

[54] Oh L.B., Teo H.H., Sambamurthy V. (2012). The effects of retail channel integration through the use of information technologies on firm performance. J. Oper. Manag..

[55] Drnevich P.L., Croson D.C. (2013). Information technology and business-level strategy: toward an integrated theoretical perspective. MIS Q..

[56] Jebran K., Chen S. (2023). Can we learn lessons from the past? COVID-19 crisis and corporate governance responses. Int. J. Finance Econ..

[57] Koutoupis A., Kyriakogkonas P., Pazarskis M., Davidopoulos L. (2021). Corporate governance and COVID-19: a literature review. Corporate Govern. Int. J. Business Soc..

[58] Rozak H., Adhiatma A., Fachrunnisa O., Rahayu T. (2023). Social media engagement, organizational agility and digitalization strategic plan to improve SMEs' performance. IEEE Trans. Eng. Manag..

[59] Beverungen D., Hess T., Köster A., Lehrer C. (2022). From private digital platforms to public data spaces: implications for the digital transformation. Electron. Mark..

[60] Kretschmer T., Leiponen A., Schilling M., Vasudeva G. (2022). Platform ecosystems as meta-organizations: implications for platform strategies. Strat. Manag. J..

[61] Teece D.J., Pisano G., Shuen A. (1997). Dynamic capabilities and strategic management. Strat. Manag. J..

[62] Lee H. (2023). Drivers of green supply chain integration and green product innovation: a motivation-opportunity-ability framework and a dynamic capabilities perspective. J. Manuf. Technol. Manag..

[63] Solberg E., Traavik L.E.M., Wong S.I. (2020). Digital mindsets: recognizing and leveraging individual beliefs for digital transformation. Calif. Manag. Rev..

[64] Subramaniam R., Singh S.P., Padmanabhan P., Gulyas B., Palakkeel P., Sreedharan R. (2021). Positive and negative impacts of COVID-19 in digital transformation. Sustainability.

[65] Tsou H.-T., Chen J.-S. (2021). How does digital technology usage benefit firm performance? Digital transformation strategy and organisational innovation as mediators. Technol. Anal. Strat. Manag..

[66] Peng Y., Tao C. (2022). Can digital transformation promote enterprise performance? —from the perspective of public policy and innovation. J. Innov. Knowl..

[67] Nasiri M., Ukko J., Saunila M., Rantala T. (2020). Managing the digital supply chain: the role of smart technologies. Technovation..

[68] Li X., Wu T., Zhang H.J., Yang D.Y. (2022). Digital technology adoption and sustainable development Performance of strategic emerging industries: the mediating role of digital technology capability and the moderating role of digital strategy. J. Organ. End User Comput..

[69] Jardak M.K., Ben Hamad S. (2022). The effect of digital transformation on firm performance: evidence from Swedish listed companies. J. Risk Finance.

[70] Gawer A. (2022). Digital platforms and ecosystems: remarks on the dominant organizational forms of the digital age. Innovation..

[71] Rocha C., Quandt C., Deschamps F., Philbin S., Cruzara G. (2023). Collaborations for digital transformation: case studies of industry 4.0 in Brazil. IEEE Trans. Eng. Manag..

[72] Gimpel H., Hosseini S., Huber R., Probst L., Röglinger M., Faisst U. (2018). Structuring digital transformation: a framework of action fields and its application at ZEISS. J. Inf. Technol. Theor. Appl..

[73] Aitken B.J., Harrison A.E. (1999). Do domestic firms benefit from direct foreign investment? Evidence from Venezuela. Am. Econ. Rev..

[74] Ilvonen I., Thalmann S., Manhart M., Sillaber C. (2018). Reconciling digital transformation and knowledge protection: a research agenda. Knowl. Manag. Res. Pract..

[75] Ko W.W., Liu G. (2015). Understanding the process of knowledge spillovers: learning to become social enterprises. Strateg. Entrep. J..

[76] Cetindamar D., Lammers T., Zhang Y. (2020). Exploring the knowledge spillovers of a technology in an entrepreneurial ecosystem—the case of artificial intelligence in Sydney. Thunderbird Int. Bus. Rev..

[77] Bouncken R., Ratzmann M., Barwinski R., Kraus S. (2020). Coworking spaces: empowerment for entrepreneurship and innovation in the digital and sharing economy. J. Bus. Res..

